# Behandlungspfade in der ambulanten Versorgung von Patienten mit Depression – Eine Routinedatenanalyse

**DOI:** 10.1055/a-2712-9615

**Published:** 2025-11-12

**Authors:** Sarah Schlierenkamp, Sandra Werner, Pauline Schlesiger, Luisa Friedrich, Carina Abels, Klemens Höfer, Dieter Best, Barbara Lubisch, Gebhard Hentschel, Franziska Weigel, Dagmar Wieczorek, Ursula Marschall, Christa Schaff, Bettina Meisel, Helene Timmermann, Anja Neumann, Anke Walendzik, Jürgen Wasem, Silke Neusser

**Affiliations:** 1 Essener Forschungsinstitut für Medizinmanagement (EsFoMed) GmbH, Essen; 2 Lehrstuhl für Medizinmanagement, Universität Duisburg-Essen, Essen; 3 Deutsche Psychotherapeutenvereinigung (DPtV), Berlin; 4 AOK Bundesverband, Berlin; 5 BARMER, Wuppertal; 6 Berufsverband für Kinder- und Jugendpsychiatrie, Psychosomatik und Psychotherapie in Deutschland e. V. (bkjpp), Mainz; 7 Vereinigung für analytische und tiefenpsychologisch fundierte Kinder- und Jugendlichen-Psychotherapie in Deutschland e.V. gegr. 1953 (VAKJP), Berlin

**Keywords:** ambulante Versorgung, Psychotherapie, Reform der Psychotherapie-Richtlinie, Routinedaten, Behandlungspfade, outpatient care, psychotherapy, reform of the psychotherapeutic directive, statutory health insurance claims data, treatment pathway

## Abstract

**Ziel:**

Im Jahr 2017 wurde die Psychotherapie-Richtlinie reformiert. Die Auswirkungen der Reform auf den Behandlungsverlauf sollen hier untersucht werden.

**Methodik:**

Es wurde ein Prä-Post-Vergleich anhand von Routinedaten durchgeführt. Analysiert wurden Behandlungspfade von Versicherten mit erstmaliger Depressionsdiagnose in der psychotherapeutischen Versorgung. Der Beobachtungszeitraum betrug jeweils ein Jahr ab Q1 2016 Prä-Zeitraum bzw. Q1 2018 Post-Zeitraum.

**Ergebnisse:**

Eingeschlossen wurden 149.941 (Prä-Zeitraum) bzw. 134.832 (Post-Zeitraum) Versicherte. Im Post-Zeitraum wurden bis zum Beginn einer Richtlinientherapie mehr Leistungen als im Prä-Zeitraum in Anspruch genommen. Die Zeit zwischen Erstkontakt und erster Therapieleistung ist im Post-Zeitraum 16,9 Tage länger als im Prä-Zeitraum.

**Schlussfolgerung:**

Die Zeit bis zum Beginn der Therapie ist nach der Reform länger geworden mit mehr in Anspruch genommenen psychotherapeutischen Leistungen. Durch die Reform stehen mehr Behandlungswege zur Verfügung, wodurch sich die Behandlungspfade stärker diversifiziert haben.

## Abstract (en)

**Objective:**

The psychotherapy Directive was reformed in 2017. The aim is to analyse the impact of the reform on treatment pathways.

**Method:**

A pre-post comparison was conducted using statutory health insurance claims data. Patients with a first diagnosis of depression in Q1 2016 (pre-period) or Q1 2018 (post-period) were included. The observation period was one year.

**Results:**

149,941 (pre-period) and 134,832 (post-period) patients were included. In the post-period, more services are used up to the start of directive-therapy than in the pre-period. The interval between first contact and first therapy service has increased by 16.9 days in the post-period.

**Conclusion:**

The time interval until the start of therapy has increased, but more psychotherapeutic services are being used. The reform means that more treatment pathways are available and that these have become more diversified.

## Hintergrund

Mit dem GKV-Versorgungsstärkungsgesetz wurde der Gemeinsame Bundesausschuss beauftragt, die Psychotherapie-Richtlinie zu reformieren 1 . Ziel der im Jahr 2017 in Kraft getretenen Reform war es, den Zugang zur Psychotherapie zu verbessern und eine zeitnahe bedarfsgerechte Versorgung zu gewährleisten 1 2 . Erreicht werden sollte dies unter anderem mit der Einführung der psychotherapeutischen Sprechstunde (pt Sprechstunde) und der psychotherapeutischen Akutbehandlung (pt Akutbehandlung).

Die Einführung der pt Sprechstunde, die grundsätzlich der Probatorik vorangestellt wird, hatte die Ziele eines niederschwelligen zeitnahen Zugangs zur ambulanten psychotherapeutischen Behandlung, der Ermittlung des Behandlungsbedarfs und der Weiterleitung in eine geeignete Versorgung 3 . Die pt Akutbehandlung sollte zudem für Patienten mit akuter psychischer Symptomatik eine zeitnahe Behandlung ermöglichen. Darüber hinaus wurden bestehende Versorgungselemente mit der Reform angepasst. So wurde die Mindestanzahl probatorischer Sitzungen, die zur Differenzialdiagnostik und zur Prüfung eines geeigneten Therapieverfahrens dienen, heruntergesetzt und die Kurzzeittherapie (KZT), die zur Richtlinientherapie zählt, in ein zweistufiges Verfahren mit 2×12 Therapiesitzungen differenziert 3 .

In Deutschland existiert ein vielfältiges Hilfe- und Gesundheitsversorgungssystem für psychisch Erkrankte, welches je nach klinischen Faktoren, Symptomschwere, Erkrankungsverlauf und Patientenpräferenz über niederschwellige psychosoziale Interventionen, medikamentöse und/oder psychotherapeutische Behandlungsalternativen verfügt 4 . Eine der häufigsten psychischen Erkrankungen ist die unipolare Depression mit einer Jahresprävalenz von ca. 8% in der erwachsenen Bevölkerung in Deutschland 5 . Eine schnelle Diagnostik und Behandlung der Erkrankten ist wichtig, um das Risiko einer Verschlechterung oder Chronifizierung zu reduzieren 6 .

Im Vorfeld der Reform wurden jedoch Zugangs- und Schnittstellenprobleme in der Versorgung psychisch Erkrankter diskutiert 7 , insbesondere der Zeitraum bis zum Beginn einer Psychotherapie 1 8 9 . Die Probleme eines zeitnahen Zugangs zur Richtlinientherapie werden auch nach der Reform diskutiert 6 . Im BARMER Arztreport wurden direkt nach der Reform vergleichsweise längere zeitliche Abstände zwischen Erstkontakt mit einem Psychotherapeuten und dem Beginn einer Richtlinientherapie festgestellt. Auch die pt Akutbehandlung ging mit langen zeitlichen Abständen zwischen dem Erstkontakt bei einem Psychotherapeuten und ersten Therapieeinheiten einher 2 . Singer et al. stellten zudem anhand von Daten aus Patientenakten von 8 psychotherapeutischen Praxen und einem Ausbildungsinstitut eine Verlängerung des Zeitraums zwischen erstem telefonischen Kontakt bis zum Therapiebeginn fest 10 . Diese Verlängerungen könnten durch die Einführung und Inanspruchnahme der pt Sprechstunde erklärt werden. Insofern stellt sich die Frage, wie sich die Einführung der neuen Elemente auf die Behandlungspfade der Versicherten und den Umfang der psychotherapeutischen Leistungen ausgewirkt haben. Diese Fragestellung wird mit der vorliegenden deskriptiven, vergleichenden Analyse auf Basis von GKV-Routinedaten adressiert. Ziel ist es, neben der Zeit bis zum Beginn einer Richtlinientherapie bzw. pt Akutbehandlung, den gesamten Behandlungsverlauf und -umfang im Prä-Post-Vergleich bei Patienten mit erstmaliger Depressionsdiagnose vergleichend zu betrachten. Zudem wird untersucht, inwieweit sich die Abstände zwischen einem Erstkontakt bei einem Psychotherapeuten (erste pt Sprechstunde bzw. erste probatorische Sitzung) und erster psychotherapeutischer Therapieleistung verändert haben. Dabei wird auch der Einfluss des Schweregrades der Depressionserkrankung berücksichtigt.

Die Analysen sind Teil des innovationsfondsgeförderten Projekts „Evaluation der Psychotherapie-Richtlinie (Eva PT-RL)“ (Förderkennzeichen: 01VSF19006). Bezogen auf die Ausgangsfragestellung des Gesamtprojekts wird mit der vorliegenden Arbeit ein Teilaspekt der Frage untersucht, ob die neuen Elemente im Vergleich zur Situation vor dem Inkrafttreten der Strukturreform zu einer Verbesserung des gesamten Behandlungs- und Versorgungsablaufs geführt haben.

## Methodik

Die Fragestellung wird mittels Prä-Post-Analyse auf Basis einer retrospektiven Kohortenstudie mit GKV-Routinedaten der Barmer und des AOK-Bundesverbandes deskriptiv betrachtet. Verglichen werden die Behandlungsverläufe von Versicherten mit erstmaliger Depressionsdiagnose vor (Prä-Zeitraum) und nach (Post-Zeitraum) der Richtlinien-Reform. Das Indexquartal (erstes Quartal des Beobachtungszeitraums) des Prä-Zeitraums ist das erste Quartal 2016 und des Post-Zeitraums das erste Quartal 2018. Der Beobachtungszeitraum beträgt 365 Tage ab dem ersten Tag des Behandlungsfalls des einzelnen Versicherten, in dem die erste Depressionsdiagnose (Indexdiagnose) im Indexquartal gestellt wurde. Das Projekt wurde von der Ethik-Kommission der Universität Duisburg-Essen positiv beschieden (Votums-Zeichen: 20-9792-BO). Es wurde eine Vollerhebung durchgeführt, um im Gesamtprojekt eine ausreichende Fallzahl für zusätzliche explorative Analysen zu gewährleisten. Eingeschlossen wurden Versicherte zwischen 21 und 90 Jahren, die im Indexquartal sowie mindestens einem weiteren Quartal innerhalb von 12 Monaten eine Depressionsdiagnose (F32, F33, F34.1, F38.1) als gesicherte und/oder Hauptentlassungsdiagnose erhalten haben. Um Versicherte mit erstmaliger Depressionsdiagnose zu selektieren, sollte in den 12 Monaten vor dem Indexquartal keine der genannten Diagnosen dokumentiert worden sein. Weitere Einschlusskriterien können dem Studienprotokoll entnommen werden 11 .

Für die Analyse wurden Versichertenstammdaten sowie ambulante Versorgungsdaten einbezogen. Relevante psychotherapeutische Leistungen wurden über EBM-Ziffern identifiziert und Abstände zwischen dem Erstkontakt bei einem Psychotherapeuten und der ersten Therapieleistung ermittelt. Zur besseren Vergleichbarkeit der Inanspruchnahme mit anderen Versorgungselementen, die in 50-minütigen Einheiten erbracht werden, wurden die Leistungen der pt Sprechstunde und der pt Akutbehandlung, die als 25-minütige Sitzungen abgerechnet werden, auf 50-minütige Sitzungen normiert. Als psychotherapeutische Leistung gelten die pt Sprechstunde, Probatorik, pt Akutbehandlung und Richtlinientherapie. Der Erstkontakt bei einem Psychotherapeuten 1 ist definiert als erste pt Sprechstunde oder Probatorik im Beobachtungszeitraum. Als Therapieleistungen zählen Leistungen der Richtlinientherapie und pt Akutbehandlung.

Die Operationalisierung des Schweregrades der Indexdiagnose (erste Depressionsdiagnose im Beobachtungszeitraum) erfolgte in Abstimmung mit den am Projekt beteiligten Psychotherapeuten: leicht (F32.0; F32.8; F32.9; F33.0; F33.8; F33.9; F34.1; F38.1), mittel (F32.1; F33.1), schwer (F32.2; F33.2), sehr schwer (F32.3; F33.3) und keine Zuordnung möglich (F33.4). Die Zuordnung der Schweregrade erfolgte hierarchisch, 1. Bei mehreren Diagnosen im Behandlungsfall wurde die schwerste Diagnose als Indexdiagnose einbezogen, 2. bei zeitgleichen Behandlungsfällen mit Depressionsdiagnosen, wurden ambulante gegenüber stationären Diagnosen priorisiert, 3. bei zeitgleichen Behandlungsfällen mit Depressionsdiagnosen durch unterschiedliche Fachgruppen, wurden ärztliche und psychologische Psychotherapeuten für Erwachsene sowie Neurologen und Psychiater (Fachgruppenschlüssel: 51, 53, 58, 59, 60, 61, 68) gegenüber anderen Facharztgruppen priorisiert, unter der Annahme, dass die Diagnostik psychischer Erkrankungen bzw. deren Abgrenzung zu somatischen Erkrankungen in der Aus- und Weiterbildung der genannten Fachgruppen einen höheren Stellenwert besitzen.

Die Abbildung der Behandlungspfade orientiert sich an der Psychotherapie-Richtlinie 12 und dem Versorgungspfad der ambulanten Versorgung des IQTIG 13 . In die Analyse der Behandlungspfade eingeschlossen wurden ausschließlich Versicherte mit exakt der angegebenen Abfolge von Leistungen. Relevante psychotherapeutische Leistungen waren die pt Sprechstunde, pt Akutbehandlung, Probatorik, KZT (Einzel und Gruppe) und Langzeittherapie (LZT) (Einzel und Gruppe). Innerhalb der Behandlungspfade wurden die Sitzungsanzahl und die Behandlungsdauer (Zeit in Tagen zwischen der ersten und letzten gleichartigen Leistung) betrachtet. Leistungen wurden nicht einbezogen, wenn mehr als 91 Tage zwischen einer Leistung und der nächsten gleichartigen Leistung lagen. Für Behandlungen, die nur eine Leistung umfassten, beträgt die Behandlungsdauer 0 Tage. Durch die neu eingeführte pt Akutbehandlung, die antragsfrei und ohne Probatorik durchgeführt werden kann 12 , ist im Post-Zeitraum die Anzahl an möglichen Behandlungspfaden höher als im Prä-Zeitraum.

Die statistischen Auswertungen erfolgten mit der Statistiksoftware SPSS Statistics Version 29. Es wurden deskriptive Analysen und Gruppenvergleiche samt geeigneter Testverfahren (z. B. Mann-Whitney-U-Test für unabhängige Stichproben) durchgeführt. Der Signifikanzwert wurde auf p≤0,05 festgelegt. Es erfolgte eine interne Qualitätssicherung der Datenaufbereitung und -auswertung. Zudem wurden entsprechend des Skalenniveaus geeignete Assoziationsmaße ermittelt. Neben der statistischen Signifikanz wurde auch die Effektstärke mittels der Koeffizienten Phi, Cramers V und Eta erfasst. Cramers V und Eta können auf einer Skala von 0 (kein Zusammenhang) bis 1 (perfekter Zusammenhang) bewertet werden, Phi kann Werte zwischen -1 und 1 annehmen. Bei den Koeffizienten Phi und Cramers V stehen V=0,1 für einen schwachen, V=0,3 für einen moderaten und V=0,5 für einen starken Zusammenhang 14 .

## Ergebnisse

Es konnten 149.941 (Prä-Zeitraum) bzw. 134.832 (Post-Zeitraum) Versicherte eingeschlossen werden ( Tab. 1 ). Die Versicherten waren durchschnittlich 54,1 bzw. 53,2 Jahre alt. In beiden Kohorten waren knapp zwei Drittel weiblich (Prä-Zeitraum: 65,3%; Post-Zeitraum: 63,6%). Der größte Anteil mit 65,6% (Prä-Zeitraum) bzw. 61,1% (Post-Zeitraum) war leicht erkrankt. Der Anteil mittelgradig Erkrankter stieg leicht von 25,2% (Prä-Zeitraum) auf 29,5% (Post-Zeitraum). Am häufigsten wurden die Indexdiagnosen in beiden Zeiträumen im ambulanten Bereich (Prä-Zeitraum: 97,2%; Post-Zeitraum: 97,4%) und dabei überwiegend durch Hausärzte gestellt (Prä-Zeitraum: 68,8%; Post-Zeitraum: 68,0%). Zudem wiesen 5,9% bzw. 5,8% Alkoholmissbrauch, 3,7% bzw. 4% Drogenmissbrauch und 3,8% bzw. 3,6% Psychosen auf.

**Table:** **Tab. 1** Soziodemographische Merkmale der eingeschlossenen Versicherten

Soziodemographische Merkmale		2016 (n=149.941)		2018 (n=134.832)		2016 vs. 2018 (n=284.773)	
		n	%	n	%	p-Wert 3	Assoziationsmaß
Alter	MW (Std.)	54,12 (±17,4)		53,18 (±17,4)		<0,001	0,054 6
Geschlecht	Weiblich	97.878	65,3%	85.714	63,6%	<0,001	0,018 5
	Männlich	52.063	34,7%	49.118	36,4%		
Ländlichkeit	Stadt	60.623	40,4%	53.408	39,6%	<0,001	0,008 4
	Mischkreis 1	50.871	33,9%	46.270	34,3%		
	Land	38.447	25,6%	35.154	26,1%		
Schweregrad 2	Leicht 7	98.316	65,6%	82.319	61,1%	<0,001	0,051 4
	Mittel 8	37.829	25,2%	39.720	29,5%		
	Schwer 9	11.226	7,5%	10.627	7,9%		
	Sehr schwer 10	1.531	1,0%	1.412	1,0%		
	Keiner zugeordnet 11	1.039	0,7%	754	0,6%		
Wo wurde die Indexdiagnose gestellt	Stationärer/ambulanter Krankenhausbereich	4.157	2,8%	3.552	2,7%	<0,5	− 0,004 5
	Ambulanter Bereich	145.784	97,2%	131.280	97,4%		
	Hausarzt	100.305	68,8%	89.276	68,0%	<0,001	0,023 4
	Psychotherapeut 12	25.881	17,8%	25.442	19,4%		
	Sonstiger Facharzt	19.598	13,4%	16.562	12,6%		
Psychische Komorbidität 13	Alkoholmissbrauch	8.863	5,9%	7.879	5,8%	n.s.	− 0,001 5
	Drogenmissbrauch	5.569	3,7%	5.382	4,0%	<0,001	0,007 5
	Psychosen	5.696	3,8%	4.862	3,6%	<0,01	− 0,005 5

^1^ Die Zuordnung nach Stadt/Land gemäß BBSR-Vorgehen basiert auf der 5-stelligen PLZ. Daher ist es möglich, dass auf der Ebene der 3-stelligen PLZ, die hier vorliegt, einige PLZ sowohl der Kategorie Stadt als auch der Kategorie Land zugeordnet sein können. Diese sind für die vorliegende Auswertung der Kategorie „Mischkreis“ zugeordnet worden;

^2^ Es wird der Schweregrad der Depressionsdiagnose zum Zeitpunkt der Indexdiagnose betrachtet;

^3^ Aufgrund der Tatsache, dass bei den Daten nicht von einer Normalverteilung ausgegangen werden konnte, wurde zur Identifizierung möglicher Gruppenunterschiede der Mann-Whitney-U-Test als nicht-parametrischer Test durchgeführt;

^4^ Da es sich um nominalskalierte Variablen handelt, wurde Cramers V berechnet;

^5^ Da es sich um binäre Variablen handelt, wurde der Phi-Koeffizient berechnet;

^6^ Da es sich um metrische Variablen handelt, wurde der Eta-Koeffizient berechnet;

^7^ ICD-10-GM: F32.0; F32.8; F32.9; F33.0; F33.8; F33.9; F34.1; F38.1;

^8^ ICD-10-GM: F32.1; F33.1;

^9^ ICD-10-GM: F32.2; F33.2;

^10^ ICD-10-GM: F32.3; F33.3;

^11^ Bei sonstiger rezidivierender depressiver Störung, gegenwärtig remittiert ICD:10-GM F33.4;

^12^ Fachgruppen, die aufgrund ihrer Ausbildung ein hohes Maß an Erfahrung mit der Differenzialdiagnostik einer Depressionsdiagnose verfügen: Nervenheilkunde/Neurologie/Psychiatrie, Neurologie, Psychiatrie/Psychiatrie und Psychotherapie, Forensische Psychiatrie, Psychosomatische Medizin und Psychotherapie, psychotherapeutisch tätige Ärzte, Psychologische Psychotherapeuten;

^13^ Kategorisierung psychischer Komorbiditäten über den gesamten Beobachtungszeitraum nach Elixhauser 21 ; MW: Mittelwert; Std.: Standardabweichung; n.s. nicht signifikant.

**Abb. 1:**
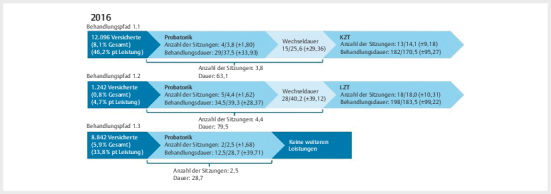
Behandlungspfade 2016. Gesamt n=149.941; Versicherte mit mindestens einer psychotherapeutischen Leistung n=26.195; über die dargestellten Pfade werden insgesamt 22.180 Versicherte abgedeckt. Das sind 14,8% der Stichprobe und 84,7% derjenigen mit mindestens einer psychotherapeutischen Leistung. Die **Behandlungsdauer** wird **in Tagen** dargestellt. Die Werte innerhalb des Behandlungspfads können wie folgt gelesen werden **Median/Mittelwert (±Standardabweichung)** . Die Werte in der Klammer darunter sind jeweils die **aufaddierten Mittelwerte** . pt: psychotherapeutisch; pt Akutbehandlung: psychotherapeutische Akutbehandlung; pt Sprechstunde: psychotherapeutische Sprechstunde.

Im Beobachtungszeitraum erhielten 17,5% (26.195 Versicherte) der Gesamtkohorte des Prä-Zeitraums und 22,8% (30.696 Versicherte) der Gesamtkohorte des Post-Zeitraums mindestens eine psychotherapeutische Leistung ( Abb. 1 2 ). Einen Erstkontakt bei Psychotherapeuten hatten 15,2% (Prä-Zeitraum: 22.806 Versicherte) bzw. 21,0% (Post-Zeitraum: 28.371 Versicherte). Mindestens eine anschließende Therapieleistung wurde bei 9,3% (Prä-Zeitraum) und 11,7% (Post-Zeitraum) abgerechnet. Wobei im Post-Zeitraum 9,7% einen Erstkontakt bei einem Psychotherapeuten mit anschließender Richtlinientherapie und 2,0% mit anschließender pt Akutbehandlung erhielten.

**Abb. 2:**
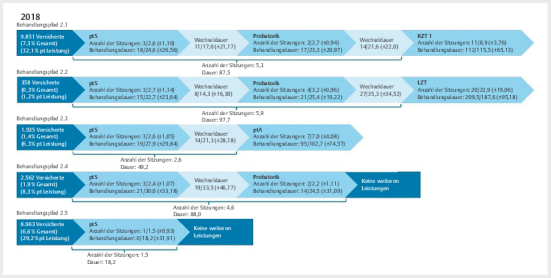
Behandlungspfade 2018. Gesamt n=134.832; Versicherte mit mindestens einer psychotherapeutischen Leistung n=30.696; über die dargestellten Pfade werden insgesamt 23.669 Versicherte abgedeckt. Das sind 17,6% der Stichprobe und 77,1% derjenigen mit mindestens einer psychotherapeutischen Leistung. Die **Behandlungsdauer** wird **in Tagen** dargestellt. Die Werte innerhalb des Behandlungspfads können wie folgt gelesen werden **Median/Mittelwert (±Standardabweichung)** . Die Werte in der Klammer darunter sind jeweils **die aufaddierten Mittelwerte** . pt: psychotherapeutisch; pt Akutbehandlung: psychotherapeutische Akutbehandlung; pt Sprechstunde: psychotherapeutische Sprechstunde.

Der durchschnittliche Abstand zwischen Erstkontakt bei einem Psychotherapeuten und erster Therapieleistung betrug 72,5 (±58,6; Prä-Zeitraum) bzw. 89,4 (±66,8; Post-Zeitraum) Tage und war damit im Post-Zeitraum statistisch signifikant länger mit einem Eta-Koeffizienten von 0,132 ( Tab. 2 ). Differenziert in Richtlinientherapie und pt Akutbehandlung betrug die durchschnittliche Dauer im Post-Zeitraum bis zur ersten Richtlinientherapie-Sitzung 94,1 (±66,3) Tage und bis zum Beginn der pt Akutbehandlung 69,4 (±68,9) Tage. Aufgeteilt nach Schweregrad der ersten Depressionsdiagnose im Beobachtungszeitraum war der zeitliche Abstand bis zur ersten Therapieleistung im Post-Zeitraum über alle Schweregrade hinweg länger als im Prä-Zeitraum. Für die Schweregrade leicht, mittel und schwer war dieser Unterschied statistisch signifikant mit Eta-Koeffizienten zwischen 0,124 (mittel) und 0,159 (schwer). Dabei ist für den Post-Zeitraum eine höhere Streuung zu verzeichnen.

**Table:** **Tab. 2** Zeitlicher Abstand zwischen Erstkontakt bei einem Psychotherapeuten und erster Therapieleistung

	2016			2018			2016 vs. 2018	
	n 1	MW (Std.)	Median (Min; Max)	n 1	MW (Std.)	Median (Min.; Max.)	p-Wert 2	Assoziationsmaß 10
Therapieleistung 3	13.962	72,50 (±58,64)	56,00 (0; 365)	15.752	89,35 (±66,77)	70,00 (0; 365)	<0,001	0,132
Richtlinientherapie	13.962	72,50 (±58,64)	56,00 (0;365)	13.104	94,12 (±66,27)	76,00 (0;365)	<0,001	0,171
pt Akutbehandlung	/	/	/	2.648	69,36 (±68,91)	45,00 (0;365)	/	/
** Abstand Erstkontakt bei einem Psychotherapeuten und erster Therapieleistung nach Schweregrad 4 der ersten Depressionsdiagnose im Beobachtungszeitraum **								
Leicht 5	6.740	72,70 (±58,22)	56,00 (0;365)	6.978	89,97 (±65,44)	71,00 (0; 365)	<0,001	0,138
Mittel 6	5.953	72,43 (±58,77)	56,00 (0;365)	7.305	88,40 (±67,43)	69,00 (0;365)	<0,001	0,124
Schwer 7	1.145	70,93 (±60,23)	53,00 (0;353)	1.329	92,11 (±69,88)	71,00 (0;359)	<0,001	0,159
Sehr schwer 8	80	80,93 (±61,99)	64,50 (14;352)	96	85,69 (±69,27)	64,50 (6;329)	n.s.	0,036
Keiner zugeordnet 9	44	71,45 (±56,48)	54,50 (5;252)	44	75,57 (±60,67)	65,50 (14;324)	n.s.	0,035

^1^ n sind hier Versicherte mit einem Erstkontakt bei einem Psychotherapeuten und mindestens einer Therapieleistung;

^2^ Aufgrund der Tatsache, dass bei den Daten nicht von einer Normalverteilung ausgegangen werden konnte, wurde zur Identifizierung möglicher Gruppenunterschiede der Mann-Whitney-U-Test als nicht-parametrischer Test durchgeführt;

^3^ Mindestens eine Leistung der pt Akutbehandlung und/oder Richtlinientherapie;

^4^ Es wird der Schweregrad der Depressionsdiagnose zum Zeitpunkt der Indexdiagnose betrachtet;

^5^ ICD-10-GM: F32.0; F32.8; F32.9; F33.0; F33.8; F33.9; F34.1; F38.1;

^6^ ICD-10-GM: F32.1; F33.1;

^7^ ICD-10-GM: F32.2; F33.2;

^8^ ICD-10-GM: F32.3; F33.3;

^9^ Bei sonstiger rezidivierender depressiver Störung, gegenwärtig remittiert ICD:10-GM F33.4;

^10^ Da es sich um metrische Variablen handelt, wurde der Eta-Koeffizient berechnet; Max.: Maximum; Min.: Minimum; MW: Mittelwert; pt Akutbehandlung: psychotherapeutische Akutbehandlung; Std.: Standardabweichung.

Mit den in Abb. 1 2 dargestellten Behandlungspfaden werden im Prä-Zeitraum 84,7% und im Post-Zeitraum 77,1% der Versicherten, die mindestens eine psychotherapeutische Leistung erhalten haben, abgebildet. Die meisten Versicherten (Prä-Zeitraum: 46,2%; Post-Zeitraum: 37,1%) beider Zeiträume erhielten eine Probatorik bzw. pt Sprechstunde plus Probatorik und anschließend eine KZT (Behandlungspfad 1.1 und 2.1). Der Weg von der ersten psychotherapeutischen Leistung bis zur ersten KZT-Sitzung betrug im Prä-Zeitraum durchschnittlich 63,1 Tage mit 3,8 Sitzungen und im Post-Zeitraum 87,5 Tage mit 5,3 Sitzungen. Daraus ergibt sich eine Behandlungsfrequenz von 16,6 Tagen im Prä-Zeitraum und 16,5 Tagen zwischen den einzelnen Leistungen im Post-Zeitraum. Die LZT als erste Therapieleistung nach der Probatorik wurde in beiden Zeiträumen deutlich seltener in Anspruch genommen als die KZT (Prä-Zeitraum: 4,7%; Post-Zeitraum: 1,2%; Behandlungspfad 1.2 und 2.2). Durchschnittlich dauerte der Behandlungspfad im Post-Zeitraum länger, allerdings wurden auch mehr Leistungen erbracht als im Prä-Zeitraum (Prä-Zeitraum: 79,5 Tage mit 4,4 Sitzungen; Post-Zeitraum: 97,7 Tage mit 5,9 Sitzungen).

Im Post-Zeitraum existiert zusätzlich der Behandlungspfad in die pt Akutbehandlung (Behandlungspfad 2.3). Diesen haben im Beobachtungszeitraum 6,3% derjenigen mit mindestens einer psychotherapeutischen Leistung in Anspruch genommen. Von der ersten pt Sprechstunde bis zur ersten pt Akutbehandlungs-Sitzung dauerte es durchschnittlich 49,2 Tage mit 2,6 Sitzungen.

Die zweithäufigsten Behandlungspfade sind Behandlungspfad 1.3 (Prä-Zeitraum) und 2.5 (Post-Zeitraum), in denen nach der Probatorik bzw. pt Sprechstunde keine weiteren Leistungen erfolgen. Im Prä-Zeitraum nahmen 33,8% und im Post-Zeitraum 29,2% derjenigen mit mindestens einer psychotherapeutischen Leistung diesen Behandlungspfad mit ca. 2,5 (Prä-Zeitraum) bzw. 1,5 (Post-Zeitraum) Sitzungen in Anspruch. Im Post-Zeitraum wurde darüber hinaus in 8,3% der Fälle die Behandlung nach der pt Sprechstunde und einer anschließenden Probatorik beendet. Hier betrug die Behandlungszeit durchschnittlich 88,0 Tage mit 4,6 Sitzungen.

Die Subgruppenanalyse nach Schweregrad der Depression für die Behandlungspfade ergab nur sehr geringfügige Unterschiede zwischen den Schweregraden innerhalb der jeweiligen Zeiträume ( **Supplement Tabelle 1** ). Der häufigste Behandlungspfad über alle Schweregrade hinweg war in beiden Zeiträumen der Pfad in die KZT (Behandlungspfad 1.1 und 2.1), am häufigsten nahmen diesen mittelgradig Erkrankte in Anspruch. Der Behandlungspfad 2.3 in die pt Akutbehandlung wurde anteilig etwas häufiger von schwer und mittelgradig Erkrankten in Anspruch genommen als von leicht Erkrankten. Unter den mittelgradig Erkrankten war zudem der Anteil derjenigen, deren Behandlungspfad nach der Probatorik (Behandlungspfad 1.3 und 2.4) bzw. der pt Sprechstunde (Behandlungspfad 2.5) beendet wurde am geringsten im Vergleich zu leicht und schwer Erkrankten.

## Diskussion

Im Index-Zeitraum 2016 erfüllten 149.941 Versicherte mit erstmaliger Depressionsdiagnose die Einschlusskriterien, im Index-Zeitraum 2018 waren es 134.832. Der durchschnittliche Abstand zwischen Erstkontakt bei einem Psychotherapeuten und erster Therapieleistung verlängerte sich nach der Reform signifikant gegenüber der Zeit vor der Reform, die Analyse ergab zudem einen schwachen bis mittleren Zusammenhang zwischen der Verlängerung und dem betrachteten Zeitraum. Die Zeit bis zum Beginn einer Richtlinientherapie war dabei durchschnittlich 21,6 Tage länger. Diese Verlängerung ging aber auch mit mehr in Anspruch genommenen Leistungen vor der Richtlinientherapie einher. Im Behandlungspfad in die KZT stieg die Anzahl von durchschnittlich 3,8 auf 5,3 Sitzungen bis zur ersten Therapiesitzung.

Der Zeitraum bis zum Beginn einer pt Akutbehandlung ist um 3,1 Tage kürzer als der Zeitraum bis zur ersten Richtlinientherapieleistung im Prä-Zeitraum. In diesem Behandlungspfad werden mit durchschnittlich 2,6 Sitzungen allerdings auch weniger Leistungen bis zum Beginn der Behandlung erbracht. Durch die pt Akutbehandlung ist mit der Reform ein neues Versorgungselement hinzugekommen, das zeitnah und antragsfrei nach der pt Sprechstunde eingesetzt werden kann, eine Probatorik zwischen pt Sprechstunde und pt Akutbehandlung ist nicht vorgesehen 12 . Wird die Dauer der Probatorik sowie die Wechseldauer zwischen pt Sprechstunde und Probatorik im Behandlungspfad in die KZT im Post-Zeitraum abgezogen, ergibt sich ein ähnlicher zeitlicher Abstand, wie im Behandlungspfad in die pt Akutbehandlung.

Insgesamt fällt für beide Zeiträume die erhebliche Schwankungsbreite in Bezug auf die zeitlichen Abstände zwischen den Leistungen und insbesondere der Wechseldauer auf. Hier fallen Median und arithmetisches Mittel auseinander und die Streuung ist hoch. Dies deutet auf eine große Heterogenität in den Verläufen hin. Allerdings ist zu berücksichtigen, dass eine klassische Normalverteilung nicht immer gegeben ist.

Im BARMER-Arztreport wird die Zeit bis zum Beginn einer Richtlinientherapie vor der Reform mit 83 Tagen und nach der Reform mit 111 Tagen angegeben. Die Zeit bis zur pt Akutbehandlung betrug 73 Tage. Der Erstkontakt ist hier definiert als erste Leistung bei einem Leistungserbringer der psychotherapeutischen Versorgung 2 . Auch Singer et al. kommen zu ähnlichen Ergebnissen. Sie ermitteln für 8 ausgewählte Praxen und eine Ausbildungsambulanz einen zeitlichen Abstand vom Erstkontakt (erste (telefonische) Kontaktaufnahme mit der Praxis) bis zum Beginn einer Richtlinientherapie von 125,3 Tagen im Prä-Zeitraum und 156,1 Tagen im Post-Zeitraum, bis zum Beginn einer pt Akutbehandlung dauert es 101,5 Tage 10 . Die Bundespsychotherapeutenkammer und die Kassenärztliche Vereinigung Bayerns kommen jeweils auf höhere zeitliche Abstände von durchschnittlich 142 bzw. 139 Tagen zwischen Erstgespräch bzw. pt Sprechstunde und Richtlinientherapie im Zeitraum nach der Reform 15 16 . In diesen Erhebungen wurde kein Prä-Post-Vergleich durchgeführt. Die Anzahl der Leistungen wurde in keiner der genannten Erhebungen in der hier dargestellten Form dem zeitlichen Abstand gegenübergestellt. Kruse et al. betrachteten in ihrer Erhebung die Inanspruchnahme von Leistungen anhand von GKV-Routinedaten und kamen ebenfalls zu dem Ergebnis, dass die Zeiten bis zum Beginn einer Richtlinientherapie länger geworden sind, mit einer hohen Inanspruchnahme von psychotherapeutischen Leistungen vor Beginn der Therapie 17 .

Hinsichtlich des Schweregrads der Depressionsdiagnose lassen sich keine Tendenzen zu einem (relativ) schnelleren Zugang zur Psychotherapie für schwer bis sehr schwer Erkrankte im Vergleich zum Zeitraum vor der Reform erkennen. Stattdessen lässt sich eine leichte Tendenz zu längeren zeitlichen Abständen für schwer Erkrankte im Vergleich zu leicht und mittelgradig Erkrankten ablesen. Dies könnte auf Zugangsprobleme in der ambulanten psychotherapeutischen Versorgung für schwer Erkrankte hindeuten, möglicherweise spielt für diese Gruppe aber auch die stationäre Versorgung eine stärkere Rolle, die hier nicht abgebildet wird.

Zu einer Veränderung der Behandlungsfrequenz scheint es nicht gekommen zu sein. Beispielsweise erfolgt im Behandlungspfad bis zur KZT sowohl vor als auch nach der Reform durchschnittlich ca. alle 16 Tage eine Leistung.

In der vorliegenden Studie wurde die pt Akutbehandlung im Verhältnis zur KZT selten in Anspruch genommen. Ähnliches berichtet auch der BARMER-Arztreport 2 . Eine Studie, die andere psychische Erkrankungen betrachtete, stellt hingegen fest, dass die pt Akutbehandlung gut angenommen wird 18 .

In der vorliegenden Studie zeigte sich in beiden Kohorten ein relevanter Anteil an Versicherten, die nach einer pt Sprechstunde bzw. Probatorik im Beobachtungszeitraum keine Therapieleistungen in Anspruch genommen haben. Werden die Gruppen mit ausschließlich Probatorik (Prä-Zeitraum) bzw. ausschließlich pt Sprechstunde (Post-Zeitraum) verglichen, zeigt sich, dass beim nicht Weiterführen der Behandlung im Post-Zeitraum durchschnittlich weniger psychotherapeutische Leistungen erbracht werden. Dies betraf alle Schweregrade, wobei in absoluten Zahlen Versicherte mit einem leichten Schweregrad deutlich häufiger betroffen waren als die übrigen Schweregrade. Dies könnte auf eine frühzeitigere Identifikation des Behandlungsbedarfs durch die pt Sprechstunde hinweisen.

Insgesamt sind Prä-Zeitraum und Post-Zeitraum nur eingeschränkt vergleichbar, da mit der pt Sprechstunde und der pt Akutbehandlung grundlegend neue Versorgungselemente eingeführt wurden, die eine Vielzahl neuer Behandlungspfade ermöglichen. Dies spiegelt sich auch darin wider, dass im Prä-Zeitraum 84,7% derjenigen mit pt Leistungen über die Behandlungspfade abgebildet werden können, im Post-Zeitraum aber nur 77,1%. Darüber hinaus erfolgten mit der Reform neue Vorgaben, etwa zur Anzahl verpflichtender probatorischer Sitzungen.

Auffällig ist, dass trotz steigender Diagnoseprävalenzen zwischen 2009 und 2017 19 die Einschlusskriterien im Prä-Zeitraum von deutlich mehr Versicherten erfüllt wurden als im Post-Zeitraum. In den Daten ist vor allem ein Rückgang an unspezifischen Diagnosen im hausärztlichen/somatischen Bereich zu erkennen, dies könnte auf ein geändertes Diagnoseverhalten hindeuten. Signifikante Unterschiede bezüglich des Alters, Geschlechts, Schweregrads der Depression und der regionalen Verteilung zwischen den Kohorten sind eher auf die große Fallzahl zurückzuführen, was sich in den geringen Assoziationsmaßen widerspiegelt.

Limitation dieser Studie ist der auf ein Jahr begrenzte Beobachtungszeitraum, durch den es zu einer Unterschätzung der Anzahl der Therapieleistungen kommen kann. Daher kann auch der übliche Behandlungspfad in die LZT über die KZT nicht vollständig abgebildet werden, was die geringe Inanspruchnahme der LZT erklären könnte. Nicht davon beeinflusst werden hingegen die Abstände zwischen dem Erstkontakt bei Psychotherapeuten und der ersten Therapieleistung, die in dieser Analyse im Fokus standen. Eine weitere Limitation ist, dass zwar Versicherte mit Depressionsdiagnosen im Jahr vor dem Indexquartal ausgeschlossen wurden, jedoch ist es möglich, dass Versicherte bereits wegen anderer Erkrankungen in psychotherapeutischer Behandlung sind oder waren. Eine gesonderte Berücksichtigung psychischer Komorbiditäten im Rahmen der Behandlungspfade fand nicht statt. Daher kann nicht ausgeschlossen werden, dass diese einen Einfluss auf die Behandlungspfade haben. Zudem ist aufgrund des eher kurzen Zeitraums von 12 Monaten vor dem Indexquartal nicht auszuschließen, dass einzelne Versicherte bereits zuvor Depressionsdiagnosen erhalten hatten. Zusätzlich ist zu berücksichtigen, dass eine eindeutige Identifikation des Zeitraums zwischen der ersten Depressionsdiagnose und dem Erstkontakt zu einem Psychotherapeuten aufgrund der Struktur der Routinedaten nicht möglich ist, da lediglich der Zeitraum des Behandlungsfalls und nicht der Stichtag der Diagnosestellung identifiziert werden kann. Daher ist es nicht möglich, die Zeit zwischen Diagnosestellung und erster pt Sprechstunde trennscharf anzugeben.

Ziel der vorliegenden Analyse war es, sowohl die Zeit bis zum Beginn einer Richtlinientherapie bzw. pt Akutbehandlung als auch den gesamten Behandlungsverlauf und -umfang im Prä-Post-Vergleich bei Patienten mit erstmaliger Depressionsdiagnose vergleichend zu betrachten. Zudem wurde untersucht, inwieweit sich die Abstände zwischen einem Erstkontakt bei einem Psychotherapeuten und erster psychotherapeutischer Therapieleistung bei verschiedenen Schweregraden der Depressionserkrankung verändert haben. Damit stellt die Untersuchung einen Teilaspekt der übergeordneten Fragestellung des Gesamtprojekts „Eva PT-RL“ zur Verbesserung des Behandlungs- und Versorgungsablaufs dar. Bezogen auf den Behandlungsverlauf und -umfang lässt sich zusammenfassend festhalten, dass sich die Zeit bis zum Behandlungsbeginn durchschnittlich erhöht hat, in diesem Zeitraum jedoch mehr psychotherapeutische Leistungen erbracht wurden. Dabei zeigen sich lediglich geringfügige Unterschiede zwischen den verschiedenen Schweregraden. Die Behandlungsfrequenz hat sich nicht verändert. Da die pt Akutbehandlung direkt im Anschluss an eine pt Sprechstunde ohne Antragsstellung erbracht werden kann, hat sie das Potenzial, einen schnelleren Therapiebeginn zu ermöglichen. Im Vergleich zur Richtlinientherapie im Prä-Zeitraum verläuft der Zugang zur Therapie über die pt Akutbehandlung auch geringfügig schneller. Jedoch spielt sie gegenüber der KZT bezüglich der Inanspruchnahme eher eine untergeordnete Rolle. Insgesamt ist eine stärkere Diversifizierung der Behandlungspfade zu beobachten. Inwieweit diese Behandlungspfade auch bedarfsgerechter sind als zum Zeitpunkt vor der Reform, kann auf Basis von Routinedaten nicht eindeutig abgebildet werden, da es nicht möglich ist, die subjektiven Präferenzen von Patienten zu analysieren. In Bezug auf die übergeordnete Fragestellung aus dem Projekt Eva PT-RL bedeutet dies, dass sich die Versorgungs- und Behandlungsabläufe von Patienten mit erstmaliger Depressionsdiagnose im Zeitraum nach der Reform verändert haben. Inwieweit diese Veränderung auch mit einer Verbesserung einher geht, kann jedoch nicht abschließend beurteilt werden und bedarf weiterer Forschung (s. dazu auch Ergebnisbericht des Gesamtprojekts „EVA PT-RL“ 20 ).

## Konsequenzen für Klinik und Praxis

- Die Reform hat den Zeitraum bis zum Therapiebeginn verlängert, allerdings werden in dieser Zeit auch mehr Leistungen erbracht.
- Die pt Akutbehandlung hat das Potenzial einen schnelleren Therapiebeginn zu ermöglichen, wird aber aktuell noch nicht häufig eingesetzt.
- Insgesamt stehen durch die Einführung der neuen Versorgungselemente mehr Behandlungsmöglichkeiten und -wege zur Verfügung, wodurch sich die Behandlungspfade stärker diversifiziert haben.

## Fördermittel

Gemeinsamer Bundesausschuss (Innovationsfonds) — 01VSF19006

## Footnotes

^1^ Es ist auch möglich, dass Versicherte vor dem Erstkontakt bei einem Psychotherapeuten bereits Therapieleistungen im Beobachtungszeitraum erhielten, diese Leistungen werden hier aber nicht als Erstkontakt gezählt, sondern ausschließlich die erste Leistung der pt Sprechstunde oder der Probatorik.

## Zusätzliches Material

Zusätzliches Material Additional Material
