# Supplementary material for: Treatment pathways in the outpatient care of patients with depression – An analysis of statutory health insurance claims data
Source: Psychiatr Prax. 2025 Nov 12;52(8):442–50. [Article in German] doi: 10.1055/a-2712-9615 (PMC12611476; doi:10.1055/a-2712-9615)
Supplement: Supplementary file 1 — Zusätzliches Material [file 10-1055-a-2712-9615-pp-2025-01-0326-oa.pdf]

Zusatzmaterial

Tabelle 1: Behandlungspfad 1.1: Probatorik – KZT (2016) nach Schweregrad[1] der ersten Depressionsdiagnose

2016 (n = 12.096)	n	% von Gesamt	% von mit pt Leistung[2]	Probatorik		Wechseldauer (Probatorik → KZT) (Md./MW(Std.))	KZT	
				Anzahl der Sitzungen (Md./MW(Std.))	Behandlungsdauer (Md./MW(Std.))		Anzahl der Sitzungen (Md./MW(Std.))	Behandlungsdauer (Md./MW(Std.))
**Leicht**	5.927	6,0%	43,9%	4/3,8 (1,82)	30/38,0 (34,17)	15/24,9 (26,89)	13/13,6 (9,12)	170/162,7 (94,64)
**Mittel**	5.084	13,4%	50,4%	4/3,8 (1,74)	29/37,1 (33,49)	16/26,4 (31,48)	14/14,9 (9,35)	199/182,6 (94,95)
**Schwer**	976	8,7%	42,3%	4/3,7 (1,89)	28/35,5 (34,54)	14/26,0 (32,55)	13/13,3 (8,39)	163/157,4 (93,51)
**Sehr schwer**	73	4,8%	36,1%	5/3,9 (1,84)	29/42,0 (38,45)	15/24,5 (24,97)	10/10,9 (7,12)	126/138,9 (100,60)
**Keiner zugeordnet**	36	3,5%	39,6%	5/3,9 (1,85)	28/35,4 (29,14)	17/24,7 (20,86)	14/14,6 (9,71)	163/159,2 (87,98)

[1] Es wird der Schweregrad der Depressionsdiagnose zum Zeitpunkt der Indexdiagnose betrachtet.
[2] Mindestens eine pt Leistung hatten 13.501 leicht, 10.094 mittelgradig, 2.307 schwer, 202 sehr schwer Erkrankte und 91 ohne zugeordneten Schweregrad.
KZT: Kurzzeittherapie; Md.: Median; MW: Mittelwert; Std.: Standardabweichung.

Tabelle 2: Behandlungspfad 1.2: Probatorik – LZT (2016) nach Schweregrad[1] der ersten Depressionsdiagnose

2016 (n = 1.242)	n	% von Gesamt	% von mit pt Leistung[2]	Probatorik		Wechseldauer (Probatorik → LZT) (Md./MW(Std.))	LZT	
				Anzahl der Sitzungen (Md./MW(Std.))	Behandlungsdauer (Md./MW(Std.))		Anzahl der Sitzungen (Md./MW(Std.))	Behandlungsdauer (Md./MW(Std.))
**Leicht**	517	0,5%	3,8%	5/4,5 (1,70)	35/41,0 (29,47)	29/40,4 (38,46)	16/16,5 (10,03)	175/167,7 (99,46)
**Mittel**	615	1,6%	6,1%	5/4,4 (1,55)	34/38,6 (27,63)	28/39,5 (38,24)	19/19,4 (10,38)	224/199,3 (97,34)
**Schwer**	102	0,9%	4,4%	5/4,2 (1,68)	31,5/35,5 (27,45)	28/41,9 (44,63)	16/17,7 (10,06)	173/170,6 (91,76)
**Sehr schwer**	<10[3]	/	/	/	/	/	/	/
**Keiner zugeordnet**	<10[3]	/	/	/	/	/	/	/

[1] Es wird der Schweregrad der Depressionsdiagnose zum Zeitpunkt der Indexdiagnose betrachtet.
[2] Mindestens eine pt Leistung hatten 13.501 leicht, 10.094 mittelgradig, 2.307 schwer, 202 sehr schwer Erkrankte und 91 ohne zugeordneten Schweregrad.
[3] Aus datenschutzrechtlichen Gründen dürfen keine geringen Fallzahlen (<10) dargestellt werden.
LZT: Langzeittherapie; Md.: Median; MW: Mittelwert; Std.: Standardabweichung.

Psychiat Prax | DOI 10.1055/a-2712-9615 | © 2025. The Author(s).

Tabelle 3: Behandlungspfad 1.3: Probatorik – keine weiteren psychotherapeutischen Leistungen (2016) nach Schweregrad[1] der ersten Depressionsdiagnose

2016 (n = 8.842)	n	% von Gesamt	% von mit pt Leistung[2]	Probatorik		
				Anzahl der Sitzungen (Md./MW(Std.))	Behandlungsdauer (Md./MW(Std.))	
Leicht	4.972	5,1%	36,8%	2/2,4 (1,67)	11/27,9 (39,03)	Keine weiteren Leistungen
Mittel	2.892	7,6%	28,7%	2/2,5 (1,69)	14/30,4 (40,86)	
Schwer	850	7,6%	36,8%	2/2,4 (1,70)	9,5/27,9 (39,65)	
Sehr schwer	95	6,2%	47,0%	2/2,3 (1,72)	7/23,6 (38,82)	
Keiner zugeordnet	33	3,2%	36,3%	2/2,2 (1,34)	7/26,8 (39,81)	

[1] Es wird der Schweregrad der Depressionsdiagnose zum Zeitpunkt der Indexdiagnose betrachtet.
[2] Mindestens eine pt Leistung hatten 13.501 leicht, 10.094 mittelgradig, 2.307 schwer, 202 sehr schwer Erkrankte und 91 ohne zugeordneten Schweregrad.
Md.: Median; MW: Mittelwert; Std.: Standardabweichung.

Tabelle 4: Behandlungspfad 2.1: pt Sprechstunde - Probatorik – KZT (2018) nach Schweregrad[1] der ersten Depressionsdiagnose

2018 (n = 9.851)	n	% von Gesamt	% von mit pt Leistung[2]	ptS		Wechseldauer (ptS → Probatorik) (Md./MW (Std.))	Probatorik		Wechseldauer (Probatorik → KZT 1) (Md./MW(Std.))	KZT 1	
				Anzahl der Sitzungen (Md./MW (Std.))	Behandlungs-dauer (Md./MW(Std.))		Anzahl der Sitzungen (Md./MW(Std.))	Behandlungs-dauer (Md./MW (Std.))		Anzahl der Sitzungen (Md./MW (Std.))	Behandlungs-dauer (Md./MW(Std.))
Leicht	4.411	5,4%	30,1%	3/2,6 (1,10)	19/26,2 (26,69)	12/17,2 (20,75)	2/2,7 (0,94)	18/23,7 (20,98)	14/21,9 (21,98)	10/8,7 (3,83)	106/111,5 (64,79)
Mittel	4.568	11,5%	35,3%	3/2,6 (1,09)	17/24,7 (25,98)	11/16,9 (21,76)	2/2,8 (0,94)	17/23,0 (20,78)	14/21,3 (21,44)	12/9,2 (3,63)	114/120,8 (65,62)
Schwer	798	7,5%	29,0%	3/2,6 (1,18)	18/27,3 (28,90)	11/16,9 (20,08)	2/2,7 (0,94)	17/23,0 (21,65)	14/21,8 (23,54)	10/8,6 (3,88)	106/108,2 (62,09)
Sehr schwer	50	3,5%	19,7%	3/2,6 (1,24)	17,5/26,5 (26,92)	13/18,3 (20,18)	2/2,6 (0,97)	14/23,9 (26,87)	16,5/32,4 (41,23)	9/7,9 (4,09)	99,5/105,0 (65,73)
Keiner zugeordnet	24	3,2%	25,8%	3/2,2 (0,90)	15/22,8 (24,83)	10/17,8 (22,40)	2/2,6 (0,92)	11/21,1 (18,81)	13,5/19,6 (14,90)	12/9,6 (4,83)	101,5/108,04 (55,85)

[1] Es wird der Schweregrad der Depressionsdiagnose zum Zeitpunkt der Indexdiagnose betrachtet.
[2] Mindestens eine pt Leistung hatten 14.669 leicht, 12.933 mittelgradig, 2.747 schwer, 254 sehr schwer Erkrankte und 93 ohne zugeordneten Schweregrad.
KZT: Kurzzeittherapie; Md.: Median; MW: Mittelwert; ptS: psychotherapeutische Sprechstunde; Std.: Standardabweichung.

Tabelle 5: Behandlungspfad 2.2: pt Sprechstunde - Probatorik – LZT (2018) nach Schweregrad[1] der ersten Depressionsdiagnose

2018 (n = 9.851)	n	% von Gesamt	% von mit pt Leistung [2]	ptS		Wechseldauer (ptS → Probatorik) (Md./MW (Std.))	Probatorik		Wechseldauer (Probatorik → LZT) (Md./MW(Std.))	LZT	
				Anzahl der Sitzungen (Md./MW (Std.))	Behandlungs-dauer (Md./MW (Std.))		Anzahl der Sitzungen (Md./MW (Std.))	Behandlungs-dauer (Md./MW (Std.))		Anzahl der Sitzungen (Md./MW (Std.))	Behandlungs-dauer (Md./MW(Std.))
**Leicht**	149	0,2%	1,0%	3/2,7 (1,04)	14/21,0 (22,96)	8/13,7 (15,05)	4/3,3 (0,92)	22/26,4 (19,16)	28/35,5 (27,78)	19/21,6 (16,75)	204/182,5 (94,33)
**Mittel**	172	0,4%	1,3%	3/2,7 (1,15)	15/23,0 (24,13)	8/14,3 (15,25)	3/3,1 (0,99)	21/23,9 (19,48)	24,5/36,7 (41,20)	22/25,4 (21,37)	220/200,7 (93,02)
**Schwer**	31	0,3%	1,1%	3/2,7 (1,14)	23/27,4 (24,01)	11/18,3 (26,28)	4/3,6 (0,88)	30/29,8 (18,26)	27/29,5 (23,01)	13/15,1 (11,71)	126/146,8 (98,96)
**Sehr schwer**	<10[3]	/	/	/	/	/	/	/	/	/	/
**Keiner zugeordnet**	<10[3]	/	/	/	/	/	/	/	/	/	/

[1] Es wird der Schweregrad der Depressionsdiagnose zum Zeitpunkt der Indexdiagnose betrachtet.
[2] Mindestens eine pt Leistung hatten 14.669 leicht, 12.933 mittelgradig, 2.747 schwer, 254 sehr schwer Erkrankte und 93 ohne zugeordneten Schweregrad.
[3] Aus datenschutzrechtlichen Gründen dürfen keine geringen Fallzahlen (<10) dargestellt werden.
LZT: Langzeittherapie; Md.: Median; MW: Mittelwert; ptS: psychotherapeutische Sprechstunde; Std.: Standardabweichung.

Tabelle 6: Behandlungspfad 2.3: pt Sprechstunde – ptA (2018) nach Schweregrad[1] der ersten Depressionsdiagnose

2018 (n = 1.935)	n	% von Gesamt	% von mit pt Leistung[2]	ptS		Wechseldauer (ptS → ptA) (Md./MW(Std.))	ptA	
				Anzahl der Sitzungen (Md./MW(Std.))	Behandlungsdauer (Md./MW(Std.))		Anzahl der Sitzungen (Md./MW(Std.))	Behandlungsdauer (Md./MW(Std.))
**Leicht**	834	1,0%	5,7%	3/2,6 (1,04)	21/29,0 (29,98)	14/21,1 (25,37)	6,25/6,9 (4,05)	95/102,4 (74,32)
**Mittel**	893	2,2%	6,9%	3/2,5 (1,05)	19/27,4 (29,71)	13/21,2 (29,79)	7/7,3 (4,08)	98/106,8 (75,68)
**Schwer**	191	1,8%	7,0%	3/2,5 (1,13)	16/25,5 (29,19)	13/23,0 (32,49)	7/6,8 /(4,21)	77/87,9 (69,56)
**Sehr schwer**	11	0,8%	4,3%	3/2,4 (1,00)	22/26,9 (25,20)	7/17,5 (23,17)	6/6,2 (4,03)	81/74,5 (50,99)
**Keiner zugeordnet**	<10[3]	/	/	/	/	/	/	/

[1] Es wird der Schweregrad der Depressionsdiagnose zum Zeitpunkt der Indexdiagnose betrachtet.
[2] Mindestens eine pt Leistung hatten 14.669 leicht, 12.933 mittelgradig, 2.747 schwer, 254 sehr schwer Erkrankte und 93 ohne zugeordneten Schweregrad.
[3] Aus datenschutzrechtlichen Gründen dürfen keine geringen Fallzahlen (<10) dargestellt werden.
Md.: Median; MW: Mittelwert; ptA: psychotherapeutische Akutbehandlung; ptS: psychotherapeutische Sprechstunde; Std.: Standardabweichung.

Psychiat Prax | DOI 10.1055/a-2712-9615 | © 2025. The Author(s).

Tabelle 7: Behandlungspfad 2.4: pt Sprechstunde – Probatorik (2018) nach Schweregrad[1] der ersten Depressionsdiagnose

**2018 (n = 2.562)**	**n**	**% von Gesamt**	**% von mit pt Leistung[2]**	**ptS**		**Wechseldauer (ptS → Probatorik) (Md./MW(Std.))**	**Probatorik**		
				**Anzahl der Sitzungen (Md./MW(Std.))**	**Behandlungsdauer (Md./MW(Std.))**		**Anzahl der Sitzungen (Md./MW(Std.))**	**Behandlungsdauer (Md./MW(Std.))**	
**Leicht**	1.274	1,5%	8,7%	3/2,4 (1,08)	21/29,5 (32,60)	16/31,7 (44,98)	2/2,2 (1,10)	14/24,0 (30,26)	**Keine weiteren Leistungen**
**Mittel**	1.023	2,6%	7,9%	3/2,4 (1,03)	21/31,0 (34,11)	20/34,4 (45,41)	2/2,2 (1,13)	14/25,6 (32,04)	
**Schwer**	246	2,3%	9,0%	3/2,3 (1,14)	21/29,1 (32,99)	21/39,9 (54,92)	2/2,1 (1,09)	13/22,6 (31,69)	
**Sehr schwer**	17	1,2%	6,7%	3/2,4 (1,08)	19/22,8 (21,78)	14/24,6 (36,41)	2/2,4 (1,27)	15/24,4 (27,18)	
**Keiner zugeordnet**	<10[1]	/	/	/	/	/	/	/	

[1] Es wird der Schweregrad der Depressionsdiagnose zum Zeitpunkt der Indexdiagnose betrachtet.
[2] Mindestens eine pt Leistung hatten 14.669 leicht, 12.933 mittelgradig, 2.747 schwer, 254 sehr schwer Erkrankte und 93 ohne zugeordneten Schweregrad.
[3] Aus datenschutzrechtlichen Gründen dürfen keine geringen Fallzahlen (<10) dargestellt werden.
Md.: Median; MW: Mittelwert; ptS: psychotherapeutische Sprechstunde; Std.: Standardabweichung.

Tabelle 8: Behandlungspfad 2.5: pt Sprechstunde –keine weiteren psychotherapeutischen Leistungen (2018) nach Schweregrad[1] der ersten Depressionsdiagnose

**2018 (n = 8.963)**	**n**	**% von Gesamt**	**% von mit pt Leistung[2]**	**ptS**		
				**Anzahl der Sitzungen (Md./MW(Std.))**	**Behandlungsdauer (Md./MW(Std.))**	
**Leicht**	4.664	5,7%	31,8%	1/1,5 (0,92)	0/17,9 (31,40)	**Keine weiteren Leistungen**
**Mittel**	3.246	8,2%	25,1%	1/1,5 (0,94)	0/18,4 (32,35)	
**Schwer**	910	8,6%	33,1%	1/1,5 (0,96)	0/19,2 (33,20)	
**Sehr schwer**	112	7,9%	44,1%	1/1,4 (0,88)	0/13,4 (26,79)	
**Keiner zugeordnet**	31	4,1%	33,3%	1/1,2 (0,89)	0/20,7 (38,95)	

[1] Es wird der Schweregrad der Depressionsdiagnose zum Zeitpunkt der Indexdiagnose betrachtet.
[2] Mindestens eine pt Leistung hatten 14.669 leicht, 12.933 mittelgradig, 2.747 schwer, 254 sehr schwer Erkrankte und 93 ohne zugeordneten Schweregrad.
Md.: Median; MW: Mittelwert; ptS: psychotherapeutische Sprechstunde; Std.: Standardabweichung.

Psychiat Prax | DOI 10.1055/a-2712-9615 | © 2025. The Author(s).
